# Supplementary material for: Multiscale dynamics of charging and plating in graphite electrodes coupling operando microscopy and phase-field modelling
Source: Nat Commun. 2023 Aug 24;14:5127. doi: 10.1038/s41467-023-40574-6 (PMC10449918; doi:10.1038/s41467-023-40574-6)
Supplement: Supplementary file 1 — Supplementary Information [file 41467_2023_40574_MOESM1_ESM.docx]

**Supplementary Information**

Multiscale Dynamics of Charging and Plating in Graphite Electrodes Coupling Operando Microscopy and Phase-field Modelling

Xuekun Lu^1,2,3,^^[[1]](#footnote-1)^*, Marco Lagnoni^4^, Antonio Bertei^4^, Supratim Das^5^, Rhodri E Owen^1,2^, Qi Li^7^, Kieran O’Regan^2,8^, Aaron Wade^1,2^, Donal P Finegan^9^, Emma Kendrick^2,8^, Martin Z Bazant^5,6^, Dan JL Brett^1,2^, Paul R Shearing^1,2,10,*^

^1^Electrochemical Innovation Lab, Department of Chemical Engineering, UCL, London, WC1E 7JE, UK

^2^The Faraday Institution, Quad One, Harwell Science and Innovation Campus, Didcot, OX11 0RA, UK

^3^School of Engineering and Materials Science, Queen Mary University of London, London, UK

^4^Department of Civil and Industrial Engineering, University of Pisa, Pisa, 56122, Italy

^5^Department of Chemical Engineering, MIT, Cambridge, MA 02139, USA

^6^Department of Mathematics, MIT, Cambridge, MA 02139, USA

^7^Department of Chemical Engineering, Beijing University of Technology, China

^8^School of Metallurgy and Materials, University of Birmingham, Birmingham B15 2TT, UK

^9^National Renewable Energy Laboratory, 15013 Denver West Parkway, Golden, CO 80401, USA

^10^Department of Engineering Science, University of Oxford, Parks Road, Oxford, OX1 3PJ

^*^Corresponding author


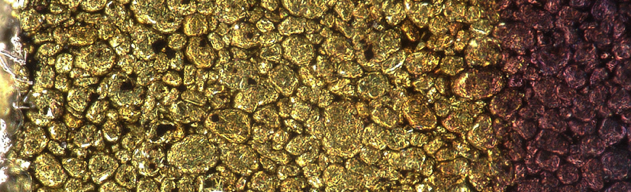

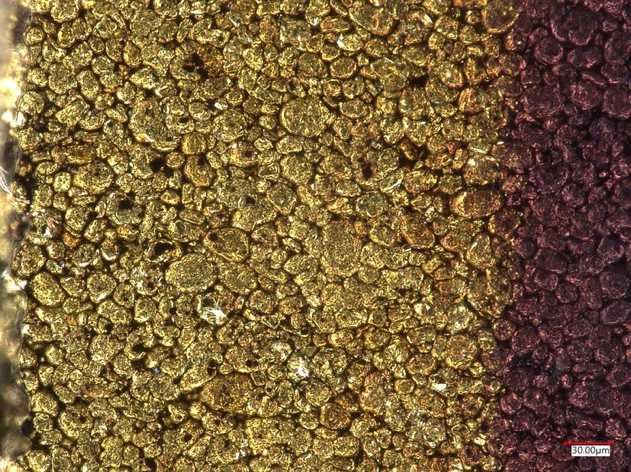

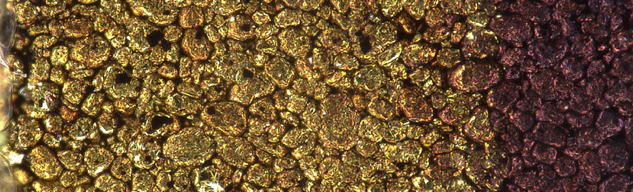


5

6


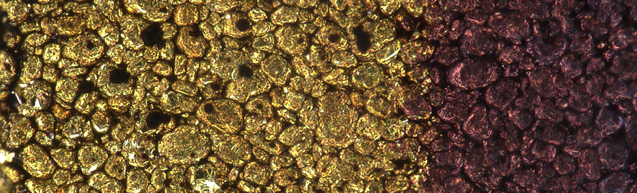

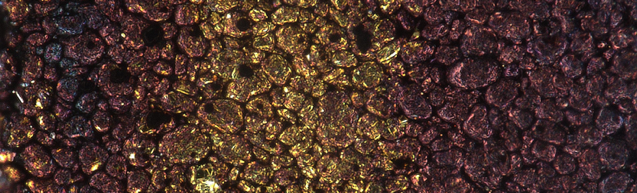

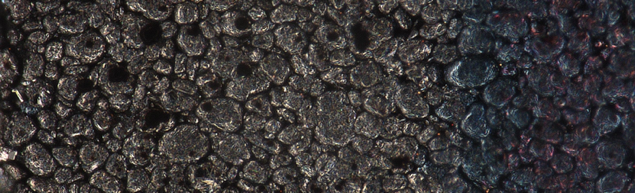

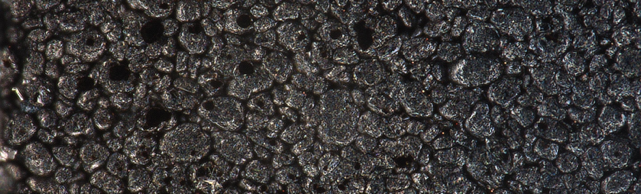


Relaxation

Discharge (4 mA cm^-2^)

18

Relaxation

15

16

17

4

Fig. S1 Optical images of Timesteps 5 and 15-18 that are not shown in the manuscript


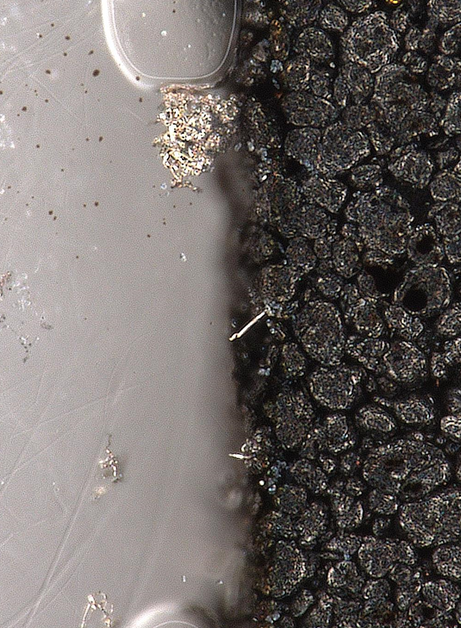


Fig. S2 Post-mortem examination of the irreversible plated lithium at the lateral surface of the electrode (LSE)


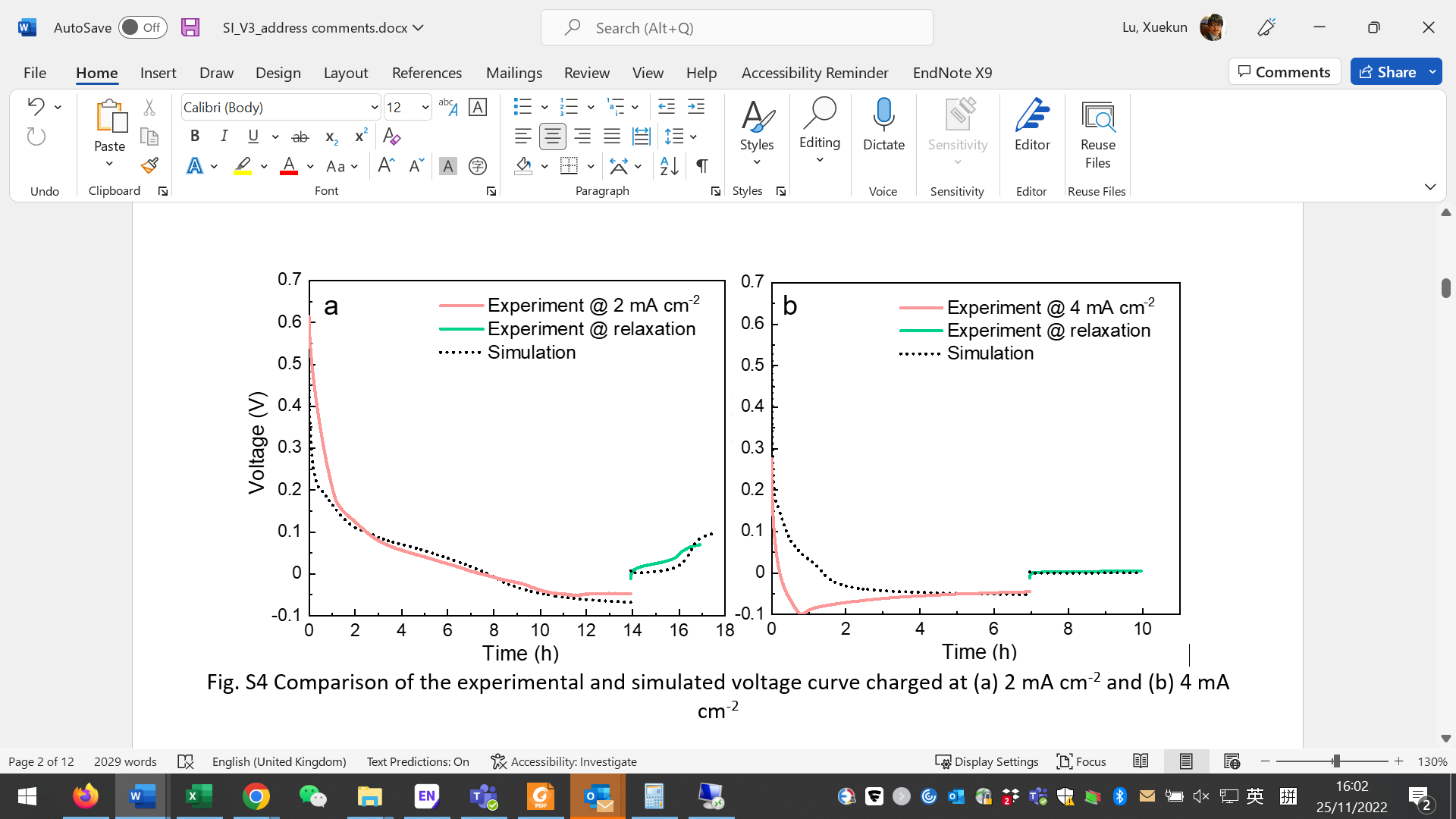


Fig. S3 Comparison of the experimental and simulated voltage curve charged at (a) 2 mA cm^-2^ and (b) 4 mA cm^-2^


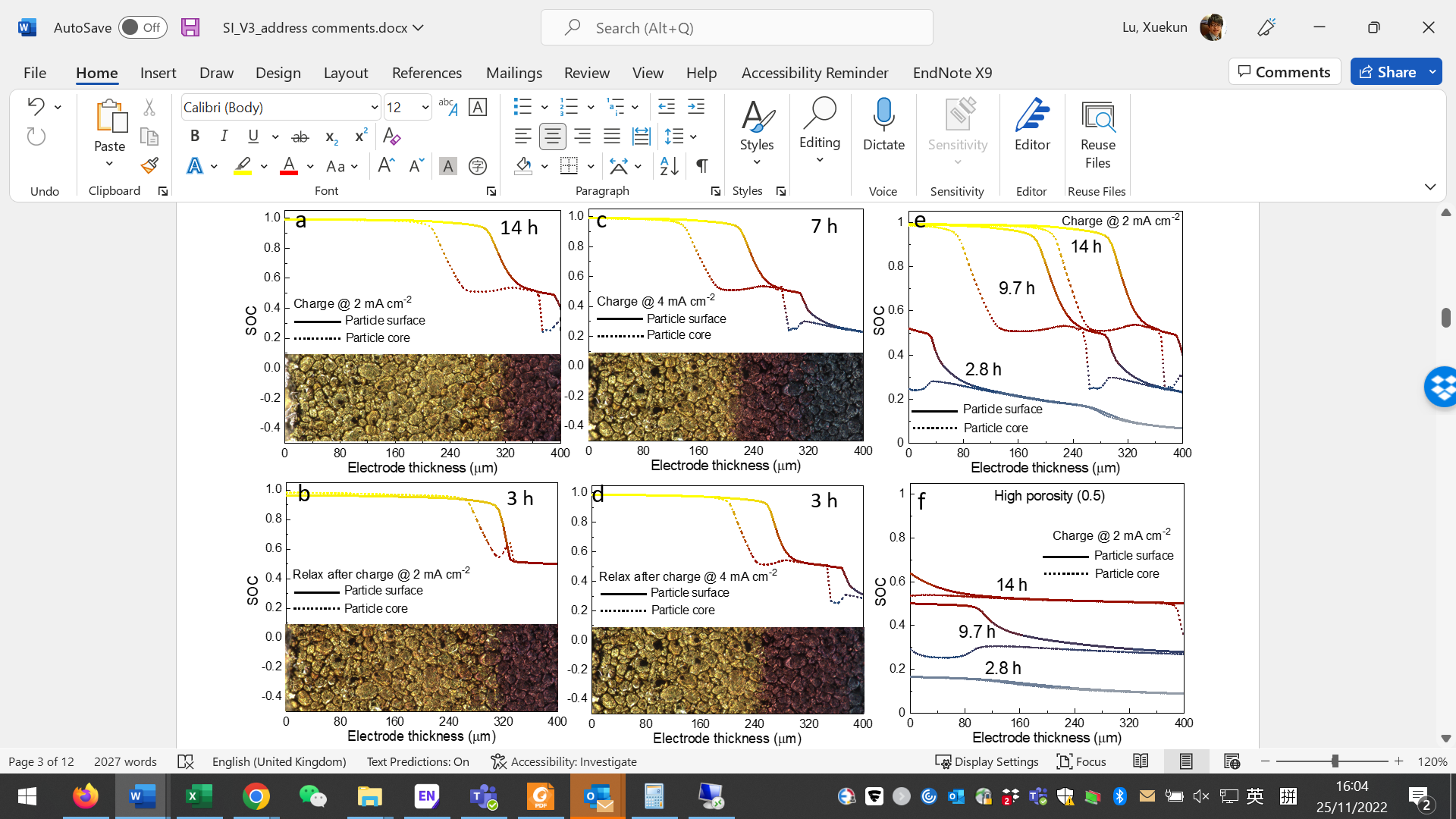


Fig. S4 The phase-separating lithiation and plating/stripping kinetics intepreted by macroscopic phase-field modelling. (a)-(b) and (c)-(d) Simulated SOC distribution after 14-hour charge and a subsequent 3-hour relaxation for 2 and 4 mA cm^-2^ charging respectively, with the curve colour-coded according to the stoichiometry vs. colour relationship of graphite particle; (e) comparison of intra-particle phase separation as a function of the incremental charging time; (f) intra-particle phase separation as a function of the incremental charging time for the high porosity (0.5) electrode


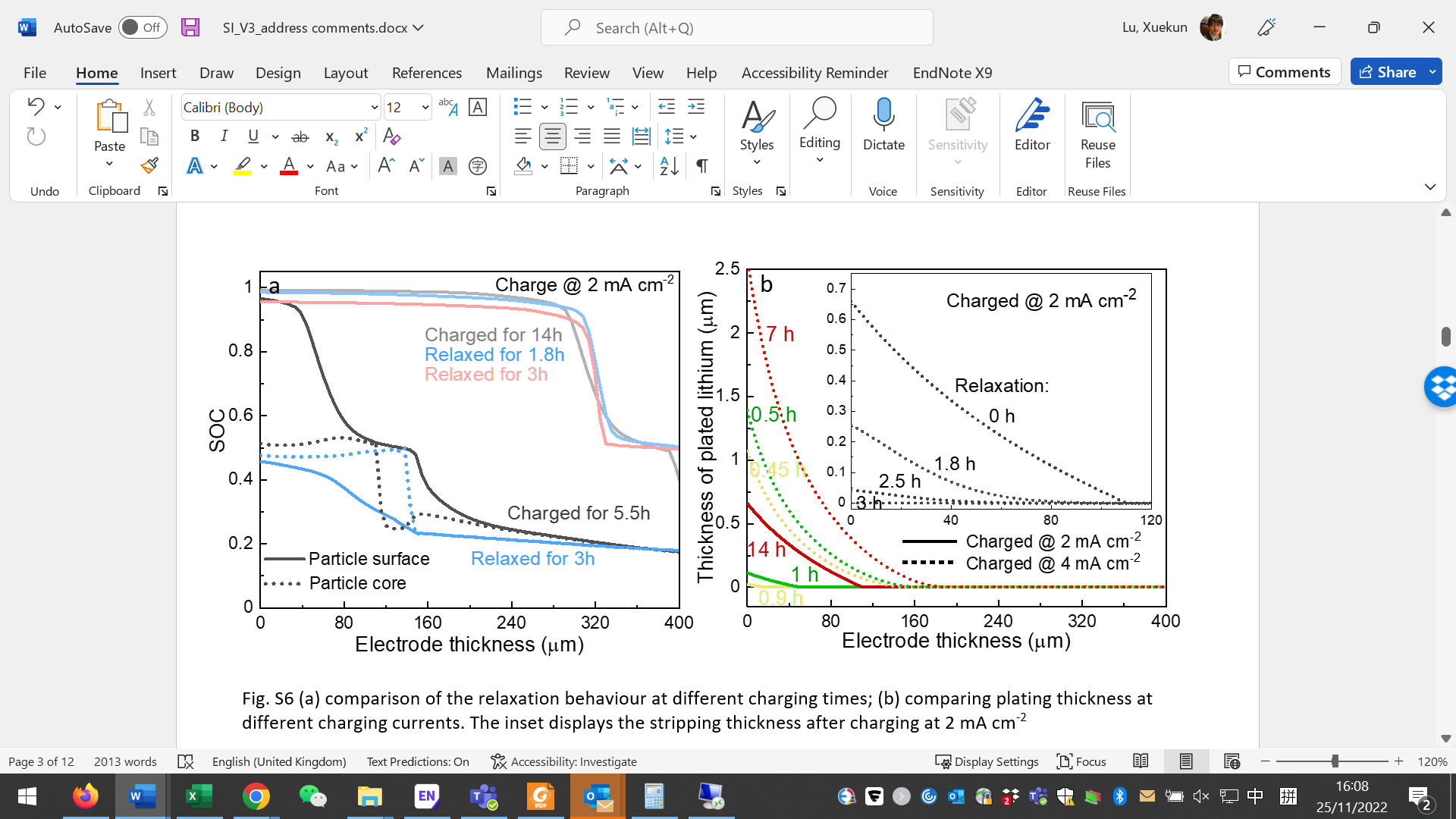


Fig. S5 Lithiation state and plating predicted by the 1D+1D phase-field model. (a) Comparison of the relaxation behaviour at different charging times; (b) comparing plating thickness at different charging currents. The inset displays the stripping thickness after charging at 2 mA cm^-2^


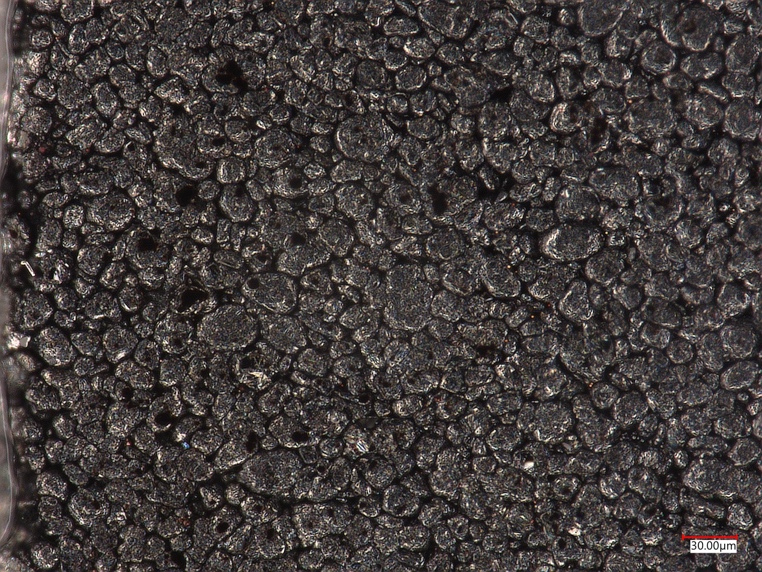


B

A

D

c

a

b

Fig. S6 Supplementary information of the operando optical characterization. (a) The selected particles investigated in Fig. 3 and (b) the relative rate of solid-state diffusion and intercalation, which indicate the transition from a reaction kinetics limited (Rp <6 μm) to a solid-state transport limited regime during lithiation at 2 mA cm^-2^


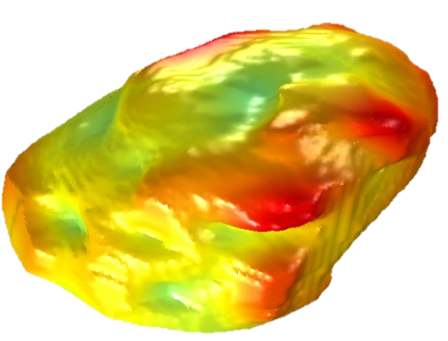


Edge

Corner


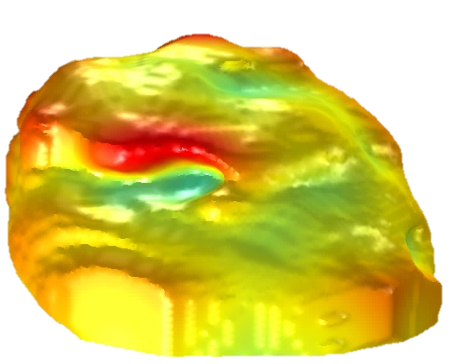


Edge

Fig. S7 Genuine representation of the edge and corner of the graphite particle

0.2

1

SOC


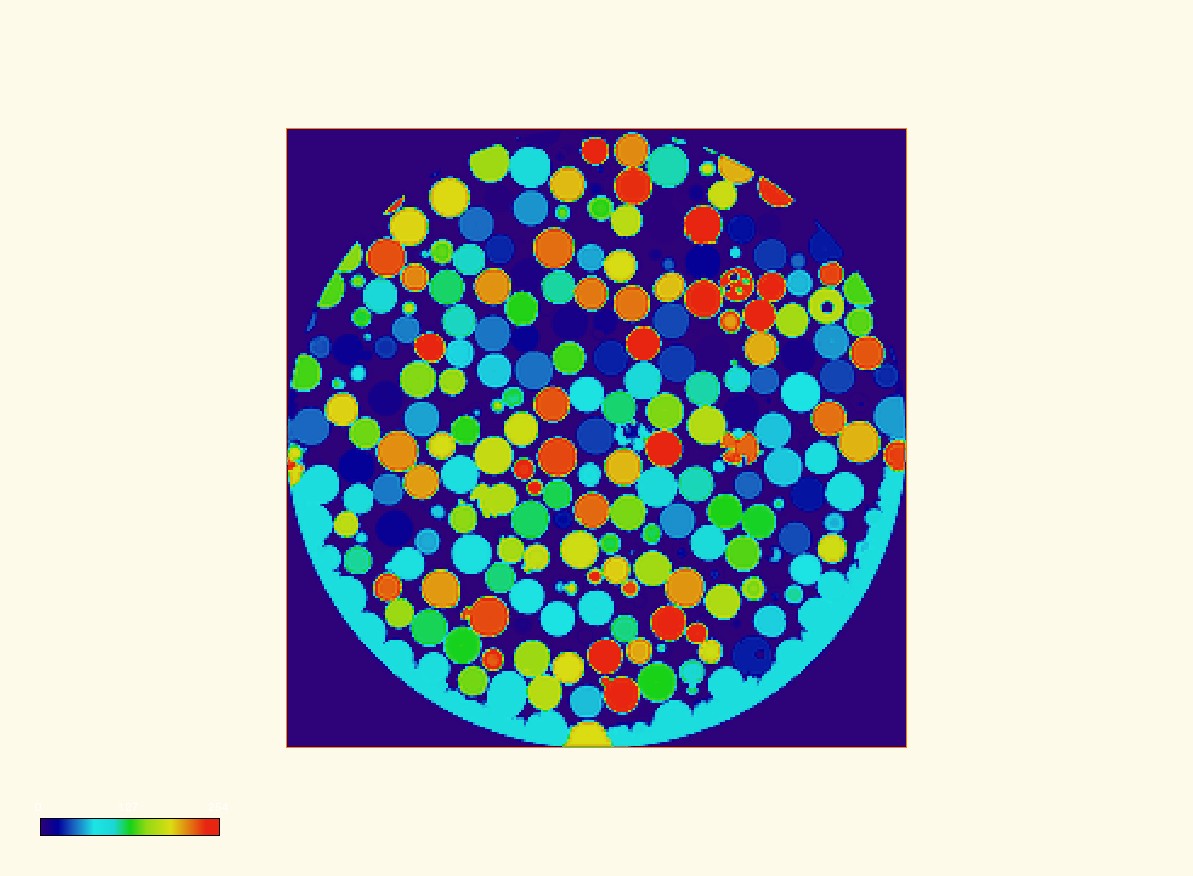

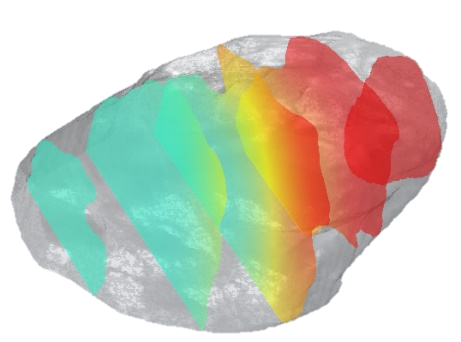

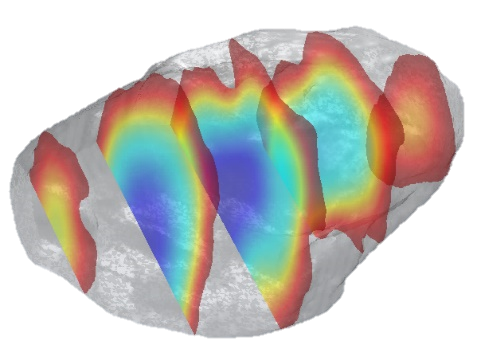


0.05C

1C

Fig. S8 Cross-sectional slices compare the difference of intercalation wave and shrinking core mechanisms charging at different C-rates


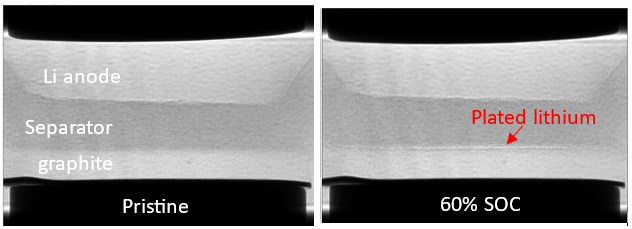


Fig. S9 Operando observation of the plating initiation in a 2 mAh cm^-2^ graphite electrode under 1C charging using synchrotron X-ray radiography (image resolution: 0.325 μm)


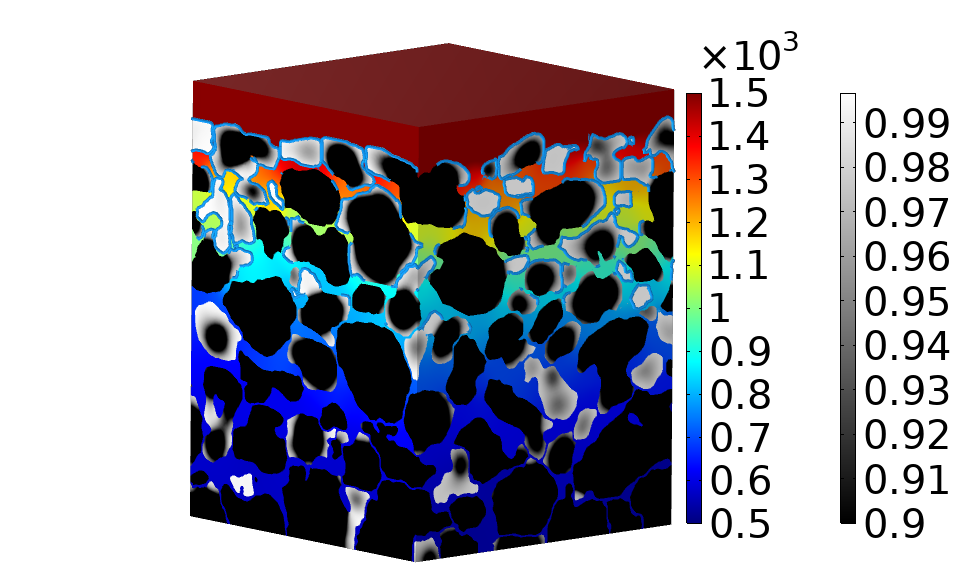


*C_e_* (mol m^-3^)

SOC

Fig. S10 Heterogeneous plating (blue lines around the particles) shown with SOC and electrolyte concentration distribution in the 4 mAh cm^-2^ electrode at 65% SOC under 1C charge. The red arrow points the local concentration inhomogeneity

a

b

Fig. S11 Electrochemical states predicted by 3D phase-field model. (a) Correlating the onset of plating, as indicated by the inflection of the SOC_avg_ curves, with the SOC_max_ and total amount of plated lithium as a function of the global SOC; (b) comparison of the experiment (solid) and simulated (dashed) voltage vs. capacity curves

Fig. S12 Solid state diffusion coefficient of lithium during intercalation in graphite particles, extracted from the literature^1^

a

b

c

d

Fig. S13 OCV relaxation profiles of the electrodes of different areal capacities after being charged at different C rates. (a) 2 mAh cm^-2^ charged at 2C; (b) 3 mAh cm^-2^ charged at 1C, (c) 2C and (d) 3C respectively

Nomenclature

| variable/parameters | |
| --- | --- |
| *J_p_* | current density associated with flux of positive ion (Li^+^), A m^-2^ |
| *J_n_* | current density associated with flux of negative ion (PF_6_^-^), A m^-2^ |
| *J_e_* | current density associated with flux of electrons (e^-^), A m^-2^ |
| *J_s_* | flux of lithium ion intercalated in the active material, A m^-2^ |
| $\mu_{p}$ | reduced electrochemical potential in ion conducting phase, V |
| $\mu_{p\_surf}$ | reduced electrochemical potential in ion conducting phase at graphite surface, V |
| $\mu_{e}$ | reduced electrochemical potential in electron conducting phase, V |
| $\tilde{\mu}$ | chemical potential in the graphite, J mol^-1^ |
| *c_e_* | concentration of the electrolyte salt, mol m^‑3^ |
| $c_{e}^{ref}$ | reference electrolyte concentration, mol m^‑3^ |
| $\tilde{c}_{e}$ | normalized electrolyte concentration (c_e_/$c_{e}^{ref}$) |
| *c_in_* | initial electrolyte concentration, mol m^‑3^ |
| *c_s_* | lithium concentration in the active material, mol m^‑3^ |
| *cmax s* | maximum lithium concentration in the active material, mol m^‑3^ |
| $\tilde{c}_{s}$ | normalized lithium concentration in the graphite (*c_s_*/*cmax s*) |
| *cin s*  *cacc s* | initial lithium concentration in the active material, mol m^‑3^  accessible lithium concentration in the active material, mol m^‑3^ |
| *t_p_* | transference number of positive ion |
| *σ_io_* | ionic conductivity in the electrolyte, S m^-1^ |
| *D_io_* | ambipolar diffusion coefficient of the binary electrolyte, m^2^ s^-1^ |
| *σ_e_* | electronic conductivity of the solid material, S m^-1^ |
| *D_s_* | solid state diffusion coefficient in active material, m^2^ s^-1^ |
| *k_ct_* | reaction rate constant of lithium intercalation, A m^-2^ |
| *i_0_* | exchange current density of lithium intercalation, A m^-2^ |
| *J_ct_* | charge transfer current density of lithium intercalation, A m^-2^ |
| *i_0_sei_* | exchange current density of SEI formation reaction, A m^-2^ |
| *J_ct_sei_* | charge transfer current density of SEI formation reaction, A m^-2^ |
| *k_ct_sei_* | reaction rate constant of SEI formation, m/s |
| *L_sei_* | SEI thickness, m |
| *c_sei_* | areal concentration of SEI layer, mol m^‑2^ |
| *M_sei_* | molar mass of SEI layer, kg mol^-1^ |
| *ρ_sei_* | mass density of SEI layer, kg m^-3^ |
| *c_ec_s_* | EC concentration at the graphite surface, mol m^‑3^ |
| *c_ec_0_* | EC concentration in the bulk electrolyte, mol m^‑3^ |
| *D_ec_* | diffusivity of EC, m^2^ s^-1^ |
| *L_film_* | thickness of the surface film, m |
| *w_sei_* | volume fraction of SEI in the film |
| *K_sei_* | conductivity of SEI layer, S m^-1^ |
| *K_Li_* | conductivity of plated lithium, S m^-1^ |
| *R_film_* | film resistance, Ω m^2^ |
| *J_ct_plt_* | charge transfer current density of lithium plating reaction, A m^-2^ |
| *i_0_plt_* | exchange current density of lithium plating reaction, A m^-2^ |
| *L_plt_* | plating thickness, m |
| *L_plt_ref_* | reference plated thickness, m |
| *c_plt_* | areal concentration of plated lithium, mol m^‑2^ |
| *M_plt_* | molar mass of plated lithium, kg mol^-1^ |
| *ρ_plt_* | mass density of plated lithium, kg m^-3^ |
| *α* | transfer coefficient for intercalation half-reaction |
| *α_sei_* | transfer coefficient for SEI formation half-reaction |
| *α_plt_* | transfer coefficient for lithium plating reaction |
| *E_b_* | energy barrier for lithium plating, V |
| *I* | applied current, A |
| *A* | cross sectional area of the electrode, m^2^ |
| *A_g_* | Specific area of the graphite, m^-1^ |
| *V_g_* | volume fraction of graphite |
| $\varepsilon^{*}$  *τ* | porosity  tortuosity in the pore phase |
| $\kappa$ | gradient energy penalty, m^2^ |
| *F* | Faraday constant, C mol^-1^ |
| *T* | temperature, K |
| *R* | gas constant, J mol^-1^ K^-1^ |
| *V_eq_* | equilibrium potential at the active material/electrolyte interface, V |
| *V_eq_sei_* | equilibrium potential of SEI formation reaction, V |

**Supplementary Method 1**

**Graphite phase-field model development (parameters with asterisk mark are for 1D+1D model only)**


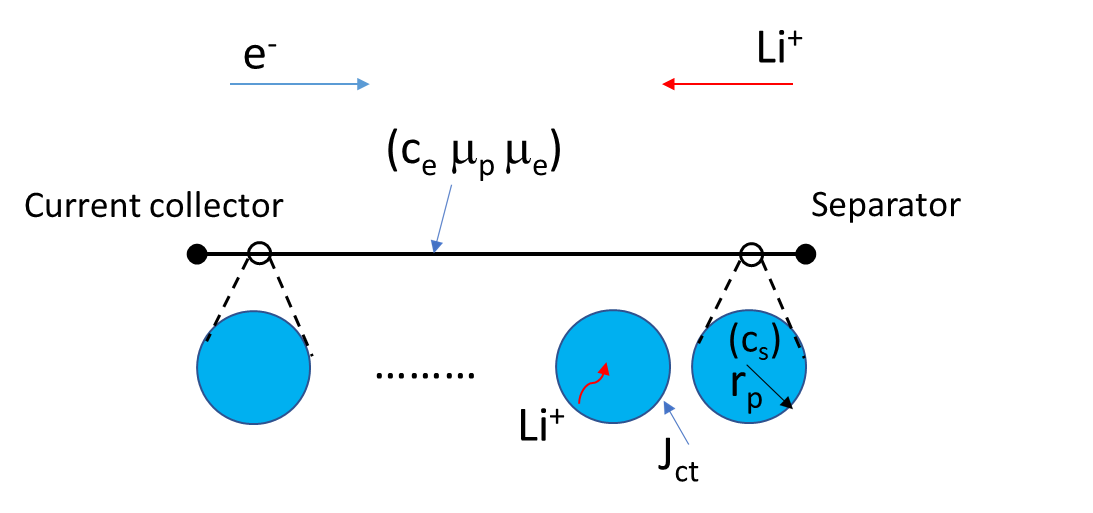

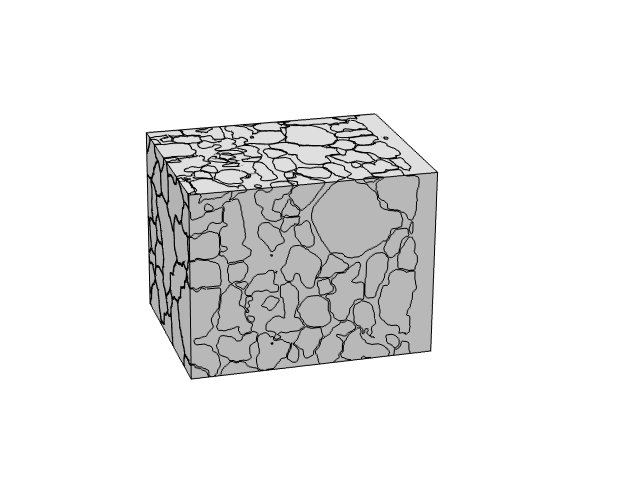


Table S1. Charge conservation and transport equations

| Phase: Electrolyte | |
| --- | --- |
| Balance equation | Flux equation |
| $\varepsilon^{*}F\frac{\partial C_{e}}{\partial t}+\nabla\cdot J_{p}=0$  $-\varepsilon^{*}F\frac{\partial C_{e}}{\partial t}+\nabla\cdot J_{n}=0$ | $J_{p}=-t_{p}{\frac{\varepsilon}{\tau}}^{*}\sigma_{io}\nabla\mu_{p}$  $J_{n}=\frac{1}{t_{p}}F{\frac{\varepsilon}{\tau}}^{*}D_{io}\nabla c_{e}-\left( 1-t_{p} \right){\frac{\varepsilon}{\tau}}^{*}\sigma_{io}\nabla\mu_{p}$ |
| Phase: Electron-conducting phase | |
| $\nabla\cdot J_{e}=0$ | $J_{e}=-\sigma_{e}\nabla\mu_{e}$ |
| Phase: Graphite particles | |
| $F\frac{\partial c_{s}}{\partial t}+\nabla\cdot J_{s}=0$ | $J_{s}=-FD_{s}/RT\cdot\left( 1-\tilde{c}_{s} \right)c_{s}\nabla\tilde{\mu}$ |
| Charge transfer kinetics | |
| $J_{ct}=i_{0}\left[ \exp(\frac{\alpha F}{RT}\left( \mu_{e}-\mu_{p\_surf}-V_{eq} \right))-\exp(-\frac{(1-\alpha)F}{RT}\left( \mu_{e}-\mu_{p\_surf}-V_{eq} \right)) \right]$ | $i_{0}=k_{ct}{\tilde{c}_{e}}^{\alpha}\tilde{c}_{s}^{1-\alpha}{(1-\tilde{c}_{s})}^{\alpha}$ |

Chemical potential $\tilde{\mu}$ in graphite particle^2^:

$$\tilde{\mu}=RT(0.18+\mu_{A}+\mu_{B}+\mu_{C}+\mu_{D}+\mu_{E}-\kappa\nabla^{2}\tilde{c}_{s})$$

where $\mu_{A}=(-40exp(-\tilde{c}_{s}/0.015)+0.75(tanh((\tilde{c}_{s}-0.17)/0.02)-1)+(tanh((\tilde{c}_{s}-0.22)/0.04)-1))\cdot SD(\tilde{c}_{s},0.35,0.05)$

$$\mu_{B}= -0.05/\tilde{c}_{s}^{0.85}$$

$$\mu_{C}= 10SU(\tilde{c}_{s},1,0.045)$$

$$\mu_{D}= 6.12(0.4-\tilde{c}_{s}^{0.98})\cdot SD(\tilde{c}_{s},0.49,0.045)\cdot SU(\tilde{c}_{s},0.35,0.05)$$

$$\mu_{E}=(1.36(0.74-\tilde{c}_{s})+1.26)\cdot SU(\tilde{c}_{s},0.5,0.02)$$

SU(x,xc,d) = 0.5(tanh((x-xc)/d)+1)

SD(x,xc,d) = 0.5 (-tanh((x-xc)/d)+1)

Voltage at equilibrium (OCV):

$$V_{eq}=E^{0}-\frac{\tilde{\mu}}{F}$$

where E^0^ is adjustable between 0.12 to 0.136 V to match the observed plateau from experiment^2^.

Balance equation and kinetics of SEI formation^3^:

$$\frac{\partial c_{sei}}{\partial t}= -\frac{J_{ct\_sei}}{2F}$$

$$J_{ct\_sei}=i_{0\_sei}\left[ \exp(\frac{\alpha_{sei}F}{RT}\left( \mu_{e}-\mu_{p\_surf}-V_{eq\_sei} \right))-\exp(-\frac{(1-\alpha_{sei})F}{RT}\left( \mu_{e}-\mu_{p\_surf}-V_{eq\_sei} \right)) \right]$$

$$i_{0\_sei}= Fk_{ct\_sei}c_{ec\_s}$$

$c_{ec\_s}$ is solved by:

$\frac{D_{ec}(c_{ec}-c_{ec\_0})}{L_{film}}$ = $\frac{J_{ct\_sei}}{F}$

SEI thickness:

$$L_{sei}=\frac{c_{sei}M_{sei}}{\rho_{sei}}$$

Balance equation and kinetics of lithium plating:

$$\frac{\partial c_{plt}}{\partial t}=-\frac{J_{ct\_plt}}{F}$$

A nucleation energy barrier of lithium plating exponentially decaying with the plating thickness is employed to describe the plating kinetics^4^:

$$J_{ct\_plt}=i_{0\_plt}\left[ \exp(\frac{\alpha_{plt}F}{RT}\left( \mu_{e}-\mu_{p\_surf}+E_{b}\cdot exp(-\frac{L_{plt}}{L_{plt\_ref}}) \right))-\exp(-\frac{(1-\alpha_{plt})F}{RT}\left( \mu_{e}-\mu_{p\_surf}+E_{b}\cdot exp(-\frac{L_{plt}}{L_{plt\_ref}}) \right)) \right]$$

Lithium plating thickness:

$$L_{plt}=\frac{c_{plt}M_{plt}}{\rho_{plt}}$$

Total film thickness:

$$L_{film}= {L_{sei}+L}_{plt}$$

Resistance of the film:

$$R_{film}= \frac{L_{film}}{w_{sei}K_{sei}+(1-w_{sei})K_{Li}}$$

$\mu_{p\_surf}$ is solved by:

$\mu_{p}- \mu_{p\_surf}= -$($J_{ct}+J_{ct\_sei}+J_{ct\_plt})R_{film}$

At the separator/electrode interface with a buffer zone (to avoid boundary effect), the external current was implemented via the current density of the positive ions in the electrolyte, whereas the negative ion current, electronic current and flux in the solid phase were set to zero. At the pore/graphite interface, the charge transfer reaction, SEI formation reaction and plating reaction affect both the flux of positive ion and the electrons, which have opposite sign. Charge transfer current density acted as the source of the flux in the solid phase. At the electrode/current collector interface, the current density in terms of positive ions, negative ions and solid phase were set to be zero, with a fixed electric potential. The initial concentration of the electrolyte and intercalated lithium in the active particles were set according to the experiment condition. The initial ionic potential was set as $-V_{eq}^{in}$ whereas the initial electric potential in the solid phase was set to 0 V, so that the difference of these two is equal to the equilibrium potential.

Table S2. Boundary conditions

| Boundary: Separator/Electrode | $n\cdot J_{n}=0$, $n\cdot J_{s}=0$, $n\cdot J_{p}=\frac{I}{A}$, $n\cdot J_{e}=0$ |
| --- | --- |
| Boundary: Pore/graphite | $n\cdot J_{p}=A_{g}^{*}(J_{ct}+J_{ct\_sei}+J_{ct\_plt})$, $n\cdot J_{n}=0$, $n\cdot J_{s}= -J_{ct}$,  $n\cdot J_{e}=-A_{g}^{*}(J_{ct}+J_{ct\_sei}+J_{ct\_plt})$ |
| Boundary: Electrode/Current collector | $n\cdot J_{p}=0$,$n\cdot J_{n}=0$, $n\cdot J_{s}=0$, $\mu_{e}=0$, |

Initial conditions:

$c_{e}=c_{e}^{in}$ , $\mu_{p}=-V_{eq}^{in}$, $c_{s}=c_{s}^{in}$, $\mu_{e}=0$

Table S3. Input parameters for different simulations

| *Parameters and domains* | | |
| --- | --- | --- |
|  | **1D+1D** | **3D** |
| ***Graphite*** |  |  |
| *D_s_*/m^2^s^-1^ | From Kühne et al. ^1^ | |
| *σ_e_*/S m^‑1^ | 50 ^5^ | |
| *cmax s*/mol m^-3^ | 29600 | |
| *cin s*/mol m^-3^ | 592 | |
| *V_g_* | 0.65 | |
| *A_g_* / m^-1^ | 3.39 $\times$10^5^ | - |
| ***Electrolyte*** |  |  |
| *D_io_*/m^2^s^-1^ | $10^{-4}\times10^{-4.43-(54/(T-229-5.0\times10^{-3}c_{e}))-0.22\times10^{-3}c_{e}}$ ^6^ | |
| *σ_io_*/S m^‑1^ | $10^{-4}\times c_{e}(-10.5+0.668\times10^{-3}c_{e}+0.494\times10^{-6}{c_{e}}^{2}$ $\text{ }+0.074T-1.78\times10^{-5}c_{e}T-8.86\times10^{-10}{c_{e}}^{2}T$ $\text{ }-6.96\times10^{-5}T^{2}+2.8\times10^{-8}c_{e}T^{2})^{2}$ ^6^ | |
| *cin e*/mol m^-3^ | 1000 | |
| *cref e*/mol m^-3^ | 1000 | |
| *t_p_* | 0.38 ^6^ | |
| *ε* | 0.28 | - |
| *τ* | 4 | - |
| ***Reaction kinetics*** |  |  |
| *k_ct_* /A m^-2^ | 1.4 ^7^ | |
| *k_ct_sei_* /m s^-1^ | 1$\times$10^-12^ ^3^ | |
| *i_0_plt_* /A m^-2^ | 0.1 ^8^ | |
| *L_plt_ref_* / m | 1$\times$10^-9^  ^4^ | |
| *α* | 0.5 | |
| *α_sei_* | 0.5 | |
| *α_plt_* | 0.5 ^4^ | |
| *E_b_* / V | 0.02 | |
| ***Physical parameters*** |  | |
| *K_sei_* / S m^-1^ | 5 $\times$10^-6^ ^3^ | |
| *K_Li_* / S m^-1^ | 1.07$\times$10^-7^ | |
| *M_sei_* / kg mol^-1^ | 0.162 ^3^ | |
| *M_plt_* / kg mol^-1^ | 6.94 $\times$10^-3^ | |
| *ρ_sei_* / kg m^-3^ | 1690 ^3^ | |
| *ρ_plt_* / kg m^-3^ | 534 | |
| *D_ec_* / m^2^ s^-1^ | 2 $\times$10^-18^ ^3^ | |
| *F* / C mol^-1^ | 96485 | |
| *R* / J mol^-1^ K^-1^ | 8.314 | |
| *T* / K | 298 | |
| $\kappa$ / m^2^ | 1 $\times$10^-11^ | |

**Supplementary Discussion 1**

Plating starts locally at the surface of graphite particles, not as a whole at the electrode scale. Thus, in this study, the nucleation energy barrier of plating (20 mV) is applied to individual graphite particles instead of a lumped overpotential value for the whole composite electrode. The rate of plating kinetics varies particle by particle, depending on the local electrochemical conditions. The nucleation energy barrier of plating (*E_b_*) is implemented as part of the calculation of the plating kinetics (shown below) locally for all the particles individually. After the plating initiates, this energy barrier decreases exponentially as the plating thickness increases. This exponential relationship was employed based on findings in Bazant’s early research^4^.

$$J_{ct\_plt}=i_{0\_plt}\left[ \exp(\frac{\alpha_{plt}F}{RT}\left( \mu_{e}-\mu_{p\_surf}+E_{b}\cdot exp(-\frac{L_{plt}}{L_{plt\_ref}}) \right))-\exp(-\frac{(1-\alpha_{plt})F}{RT}\left( \mu_{e}-\mu_{p\_surf}+E_{b}\cdot exp(-\frac{L_{plt}}{L_{plt\_ref}}) \right)) \right]$$

*E_b_* was determined by matching the predicted phase and SOC distributions (indicated by the optical colour) of Li intercalation along the horizontal thickness direction with the operando observation at the end of charge and relaxation for different charging current densities (Fig. S4). If *E_b_* is over-estimated, plated lithium is then underestimated, meaning an over-estimation of the intercalated Li, indicated by the shift of the phase boundaries and SOC distribution towards the right-hand side of the field-of-view; accordingly, during relaxation, the propagation of the phase boundary (e.g. the gold/red boundary in Fig. S4c and d) arising from re-intercalation of the plated lithium is less remarkable due to the underestimated plating. The opposite applies if *E_b_* is underestimated. We found that *E_b_* = 20 mV provides the optimal consistency with the experiment. The predicted relaxation voltage curve shows a more extended stripping process than the experiment (green curve in Fig. S3a), which might be because the model does not account for the dead lithium formed during stripping, and regard all the plated lithium as reversible.

The macroscopic phase-field model complements the optical operando experiment in revealing the intra-particle SOC distribution. Fig. S5e shows that the intra-particle phase-separation of Stage 2/3 initiates from the lateral surface of the electrode (LSE) after 2.8 h charge, followed by the coexistence of Stage 1/2/3 after 9.7 h charge. To highlight the influence of electrolyte concentration gradient in the thickness direction, a virtual high-porosity (0.5) electrode is modelled (Fig. S5f) and compared with the original electrode (Fig. S5e, porosity = 0.28). No phase separation nor three-stage coexistence along the thickness direction is found after 2.8 h charge (for the high porosity simulation). The improved electrolyte transport property due to high porosity promotes homogeneous intercalation and significantly suppresses SOC saturation at the LSE, which effectively mitigates the plating risk.

Fig. S6a highlights the distinct relaxation behaviour at different SOCs, taking 2 mA cm^-2^ case as an example. After 14 hours lithiation to a global SOC of 80%, the Stage 1/2 phase boundary becomes sharper as the relaxation time increases, attributed to the equilibration of the unstable lithium concentration. Note that a conventional solid-solution model is not able to capture the phase-separation behaviour, and thus a homogenized SOC distribution will be predicted, leading to an underestimation of the plating risk. Differing from what is observed at high SOCs, relaxation behaviour is different at an intermediate SOC (i.e., after 5.5 h charge) when the particle surface is unstable (black solid line), and the particle core is stable (black dotted line); the lithium equilibration takes place in terms of intra and inter-particle lithium exchange, causing a sharp drop in the surface concentration. This phenomenon implies that a rest step at intermediate SOC embedded in a fast charge protocol could be more conducive to plating mitigation compared to that at high SOC, as will be further elaborated in the last section of the manuscript.

**References**

1 Kühne, M. *et al.* Ultrafast lithium diffusion in bilayer graphene. *Nature Nanotechnology* **12**, 895-900, doi:10.1038/nnano.2017.108 (2017).

2 Smith, R. B., Khoo, E. & Bazant, M. Z. Intercalation Kinetics in Multiphase-Layered Materials. *The Journal of Physical Chemistry C* **121**, 12505-12523, doi:10.1021/acs.jpcc.7b00185 (2017).

3 Yang, X.-G., Leng, Y., Zhang, G., Ge, S. & Wang, C.-Y. Modeling of lithium plating induced aging of lithium-ion batteries: Transition from linear to nonlinear aging. *Journal of Power Sources* **360**, 28-40, doi:<https://doi.org/10.1016/j.jpowsour.2017.05.110> (2017).

4 Gao, T. *et al.* Interplay of Lithium Intercalation and Plating on a Single Graphite Particle. *Joule* **5**, 393-414, doi:<https://doi.org/10.1016/j.joule.2020.12.020> (2021).

5 Thomas-Alyea, K. E., Jung, C., Smith, R. B. & Bazant, M. Z. In Situ Observation and Mathematical Modeling of Lithium Distribution within Graphite. *Journal of The Electrochemical Society* **164**, E3063-E3072, doi:10.1149/2.0061711jes (2017).

6 Cai, L. & White, R. E. Mathematical modeling of a lithium ion battery with thermal effects in COMSOL Inc. Multiphysics (MP) software. *Journal of Power Sources* **196**, 5985-5989, doi:<https://doi.org/10.1016/j.jpowsour.2011.03.017> (2011).

7 Chen, C.-H. *et al.* Development of Experimental Techniques for Parameterization of Multi-scale Lithium-ion Battery Models. *Journal of The Electrochemical Society* **167**, 080534, doi:10.1149/1945-7111/ab9050 (2020).

8 Carelli, S. & Bessler, W. G. Prediction of Reversible Lithium Plating with a Pseudo-3D Lithium-Ion Battery Model. *Journal of The Electrochemical Society* **167**, 100515, doi:10.1149/1945-7111/ab95c8 (2020).

1. * Corresponding email: [xuekun.lu@qmul.ac.uk](mailto:xuekun.lu@qmul.ac.uk) (Xuekun Lu), [p.shearing@ucl.ac.uk](mailto:p.shearing@ucl.ac.uk) (Paul R Shearing) [↑](#footnote-ref-1)
