# Supplementary material for: Multiscale dynamics of charging and plating in graphite electrodes coupling operando microscopy and phase-field modelling
Source: Nat Commun. 2023 Aug 24;14:5127. doi: 10.1038/s41467-023-40574-6 (PMC10449918; doi:10.1038/s41467-023-40574-6)
Supplement: Supplementary file 3 — Description of Additional Supplementary Information [file 41467_2023_40574_MOESM3_ESM.pdf]

Description of Additional Supplementary Files

Supplementary Movie 1: Operando optical visualization of the complete charging-relaxationdischarge cycle in the graphite electrode.
